# *Akkermansia muciniphila* impacts group B *Streptococcus* vaginal colonization

**DOI:** 10.1128/mbio.02868-25

**Published:** 2026-04-27

**Authors:** Stephanie M. Marroquin, Shirli Cohen, Melody N. Neely, Kelly S. Doran

**Affiliations:** 1 Department of Immunology and Microbiology, University of Colorado School of Medicine 12225, Aurora, Colorado, USA; 2 Department of Molecular and Biomedical Sciences, The University of Maine 6250, Orono, Maine, USA; Universite de Geneve, Geneva, Switzerland

**Keywords:** group B *Streptococcus*, *Akkermansia muciniphila*, vaginal colonization, microbial interactions, RNA-seq, probiotic

## Abstract

*Streptococcus agalactiae,* or group B *Streptococcus* (GBS), is an opportunistic pathogen that asymptomatically colonizes the vaginal tract of up to 30% of healthy individuals. However, during pregnancy, it is associated with adverse pregnancy outcomes, and GBS can be transmitted to the fetus *in utero* or the newborn during vaginal birth, resulting in invasive neonatal disease. Previously, we identified that *Akkermansia muciniphila* increases GBS vaginal persistence in a cohort of human vaginal microbiome samples collected throughout pregnancy and promotes GBS vaginal colonization in a murine model. However, the mechanisms responsible for these observations are unknown. Here, we analyze additional vaginal shotgun metagenomic data sets and show that across independent studies with diverse populations, *A. muciniphila*-positive samples had higher GBS abundance. We determined that *A. muciniphila* aggregates with human vaginal isolates of GBS across all serotypes and promotes GBS attachment to human vaginal epithelial cells (hVECs). RNA-sequencing analysis reveals that *A. muciniphila* changed the expression of 281 unique GBS genes during hVEC co-colonization, many of which are involved in cell wall/membrane/envelope biogenesis. We demonstrate the importance of the GBS capsule and pili for direct interaction with *A. muciniphila* and increased attachment to hVECs, respectively. Lastly, we found that *A. muciniphila* promoted GBS aggregation in the murine vaginal lumen and that continual treatment with *A. muciniphila* reduced GBS vaginal persistence. Our results provide mechanistic insights and further evidence of the impact of *A. muciniphila* on GBS vaginal colonization and also demonstrate a beneficial potential of *A. muciniphila* treatment in the vaginal environment.

**IMPORTANCE:**

Group B *Streptococcus* (GBS) is a frequent colonizer of the vaginal tract of healthy people; however, during pregnancy, maternal colonization is associated with adverse pregnancy outcomes. GBS is a leading cause of neonatal sepsis and meningitis, with transmission to neonates occurring either during vaginal delivery or through ascension into the uterus during pregnancy. The influence of the vaginal microbiota on GBS pathogenesis remains greatly underappreciated. We have found that GBS is associated with the mucin-degrading intestinal commensal *Akkermansia muciniphila*, a newly identified colonizer of the vaginal tract. Our research identifies the mechanistic impact of this commensal organism on GBS aggregation, cell adherence, and gene expression, as well as its therapeutic potential during GBS vaginal colonization. Unraveling relationships between GBS and the vaginal microbiota will improve maternal-fetal health and may facilitate the development of alternative methods to reduce GBS *in utero* complications and neonatal disease.

## INTRODUCTION

*Streptococcus agalactiae*, or group B *streptococcus* (GBS), is a Gram-positive, β-hemolytic opportunistic pathogen that asymptomatically colonizes the female genital tract (FGT) and gastrointestinal (GI) tract in 25–30% of healthy women (1). During pregnancy, GBS is associated with adverse pregnancy outcomes, including preterm premature rupture of membranes (PPROM), chorioamnionitis, stillbirth, and preterm birth (2–4). Notably, most preterm births are due to ascending microbial infections, with 10% of these being caused by GBS (5). Maternal GBS GI and/or vaginal tract colonization is the primary risk factor in neonatal GBS disease, and approximately 50% of GBS-colonized mothers deliver newborns who are also colonized with GBS (6, 7). This transmission of GBS can subsequently lead to neonatal pneumonia, sepsis, and meningitis (8). GBS encodes for a myriad of virulence factors, including cell envelope-associated factors, such as pili, serine-rich repeat (Srr) proteins, and capsular polysaccharides (CPS), which contribute to host cell interaction and immune evasion (5, 9–13). One of the most notable GBS virulence factors is the CPS, which is uniquely sialylated and provides GBS with the capacity for “molecular mimicry” within the host (14). To date, 10 capsular serotypes have been identified, with Ia, Ib, II, III, and V being the most associated with disease worldwide (1). Importantly, a large proportion of neonatal meningitis is caused by serotype III and sequence type (ST)−17 strains (15, 16).

During pregnancy, GBS FGT colonization may be intermittent and transient (17). This is likely a consequence of GBS determinants, the commensal microbiota, and host immune responses (18). In the vagina, GBS must overcome various challenges to successfully colonize the host, including the physical barrier created by mucins on epithelial cells, competition with resident microbiota, and mucosal immunity (19, 20). Importantly, the vaginal microbiota is composed of numerous taxa that vary greatly based on factors, such as geography, race or ethnicity, hormone cycles, and pregnancy; thus, the relationship between GBS and the microbiota is complex (21). Recently, a study from our laboratory identified *Akkermansia* species in the murine vaginal microbiota and found that *Akkermansia muciniphila* promoted GBS vaginal colonization (22); however, the mechanisms underlying these observations are not known. Further examination of human vaginal samples from a pregnancy cohort identified the co-occurrence of GBS and *A. muciniphila* in the vaginal tract, suggesting that *A. muciniphila* may impact the human FGT (22). However, there has been little investigation of *A. muciniphila* in the vagina or its association with GBS.

*A. muciniphila* is a Gram-negative, mucin-degrading anaerobe that was isolated from human feces in 2004 and can be found in approximately 90% of healthy individuals (23, 24). Identified as an intestinal symbiont colonizing the mucosal layer, *A. muciniphila* has been extensively studied for its role in the GI tract, and its pilin (Amuc_1100) has been shown to contribute to host immunological homeostasis at the gut mucosa (25–27). These studies have driven the popularity of *A. muciniphila* as a probiotic supplement, with numerous private companies now selling oral probiotic supplements containing either live *A. muciniphila* or pasteurized *A. muciniphila* (28, 29). Importantly, previous research has also shown that *A. muciniphila* strains can vary in oxygen sensitivity, with Muc^T^ (our strain of interest) being significantly aerotolerant (30). Here, we examine the mechanisms by which *A. muciniphila* and GBS interact to influence vaginal colonization and identify key GBS surface factors that mediate interactions with *A. muciniphila* and the host. Our findings demonstrate the importance of *A. muciniphila* on GBS pathogenesis and highlight the complexity of interactions between commensal organisms and opportunistic pathogens in the vaginal niche.

## RESULTS

### GBS and *A. muciniphila* co-occur in the human vaginal tract across diverse populations

**Fig 1:**
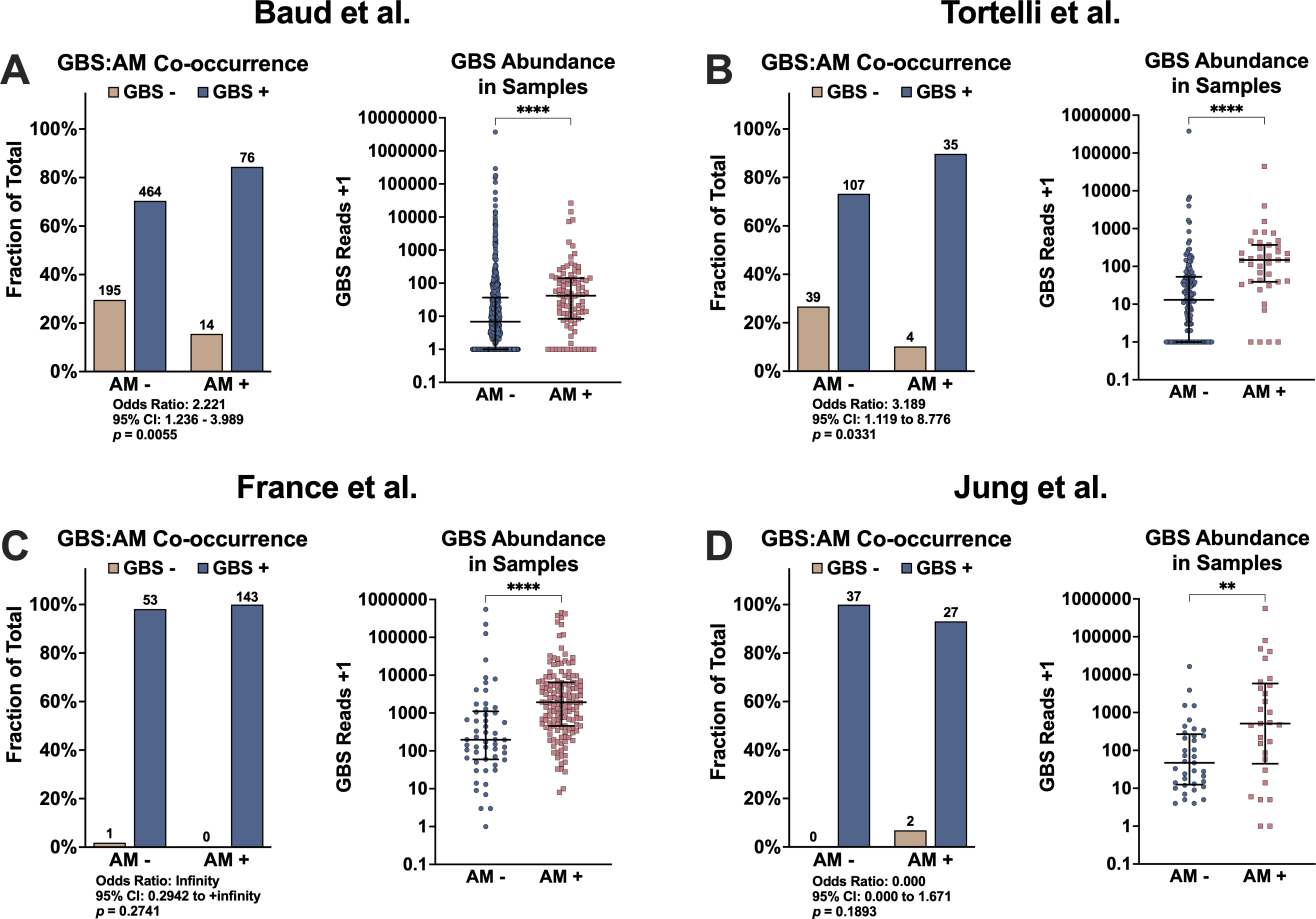
Co-occurrence of GBS and *A. muciniphila* in the human vaginal tract. Co-occurrence of GBS in *A. muciniphila* negative (AM−) and positive (AM+) (**A and B**) pregnant and (**C and D**) non-pregnant individuals. Values above the bar indicate the raw number of samples in each group. GBS abundance, represented by GBS reads in scatter dot plots, shows the median with interquartile range. Statistical analysis was determined by Fisher’s exact test with Baptista-Pike method for co-occurrence and Mann-Whitney *U* test for abundance. Data points represent study individuals. **, *P* ≤ 0.01; ****, *P* ≤ 0.0001.

To assess the presence of *A. muciniphila* in the human vaginal tract, we examined four publicly available shotgun metagenomic data sets. Two data sets originated from metagenomic studies of pregnant individuals (Baud et al. and Tortelli et al.) and two data sets originated from metagenomic studies of non-pregnant individuals (France et al. and Jung et al.) (31–34). In Baud et al., data were derived from vaginal swabs of pregnant individuals at time of birth from three Parisian hospitals (no additional race/ethnicity demographics provided) (31). Our analysis demonstrated that 12% of samples were positive for *A. muciniphila* (AM+), and of these AM+ samples, 84.4% were also positive for GBS (GBS+) (odds ratio: 2.221, 95% CI: 1.236–3.989, *P* = 0.0055). Upon sorting GBS sequencing reads by the presence or absence of *A. muciniphila*, AM+ samples showed a 0.6-log increased abundance of GBS than samples that were negative for *A. muciniphila* (AM−) (median GBS reads: 41.7 vs 6.9) (Fig. 1A). In Tortelli et al., data originated from cervicovaginal swabs of pregnant individuals (age: median = 29; IQR = 25–33) across various points of gestation, and this cohort was North American (50% White, 29% Hispanic, 10% Black, 7% Asian, 4% Other) (32). Our analysis demonstrated that *A. muciniphila* was present in 21% of samples, and that 89.7% of samples that were AM+ were also GBS+ (odds ratio: 3.189, 95% CI: 1.119–8.776, *P* = 0.0331). Examination of GBS reads between AM+ and AM− groups demonstrated a 1.1-log increase in abundance when *A. muciniphila* was present (median GBS reads: 148 vs 13) (Fig. 1B).

In France et al., metadata were obtained from vaginal swabs of non-pregnant reproductive-age individuals (age: 19–45), and this cohort was North American (62% Black, 26% White, 10% Hispanic/Latino, 3% Asian) (33). Here, we found that 72% of samples were AM+, and of those samples, 100% were GBS+. Because all AM+ samples were GBS+ in this cohort, the odds ratio is formally finite, and no statistical significance was found by Fisher’s exact test (odds ratio: infinity, 95% CI: 1.119–8.776, *P* = 0.2741). However, GBS abundance was significantly increased in AM+ samples compared to AM− samples by nearly 1 log (median GBS reads: 1921 vs 197) (Fig. 1C). In Jung et al., data originated from cervicovaginal swabs of non-pregnant individuals with histologically validated cervical lesions, and this cohort was from South Korea (no additional race/ethnicity demographics provided) (34). Our analysis demonstrated that 44% of samples were AM+, and 93.1% of these samples were also GBS+, but no statistically significant difference in the odds of being GBS+ was determined between the AM+ and AM− groups (odds ratio: 0.000, 95% CI: 0.000–1.671, *P* = 0.1893). Nonetheless, a significant 1.1-log increase was observed when examining abundance of GBS in AM+ compared to AM− samples (median GBS reads: 511 vs 47) (Fig. 1D). Altogether, these data indicate that GBS and *A. muciniphila* co-occur in these cohorts and that abundance of GBS is significantly higher in individuals that are AM+ independent of pregnancy and across diverse populations.

### GBS and *A. muciniphila* co-aggregate *in vitro* and exhibit increased attachment to human vaginal epithelial cells (hVECs)

**Fig 2:**
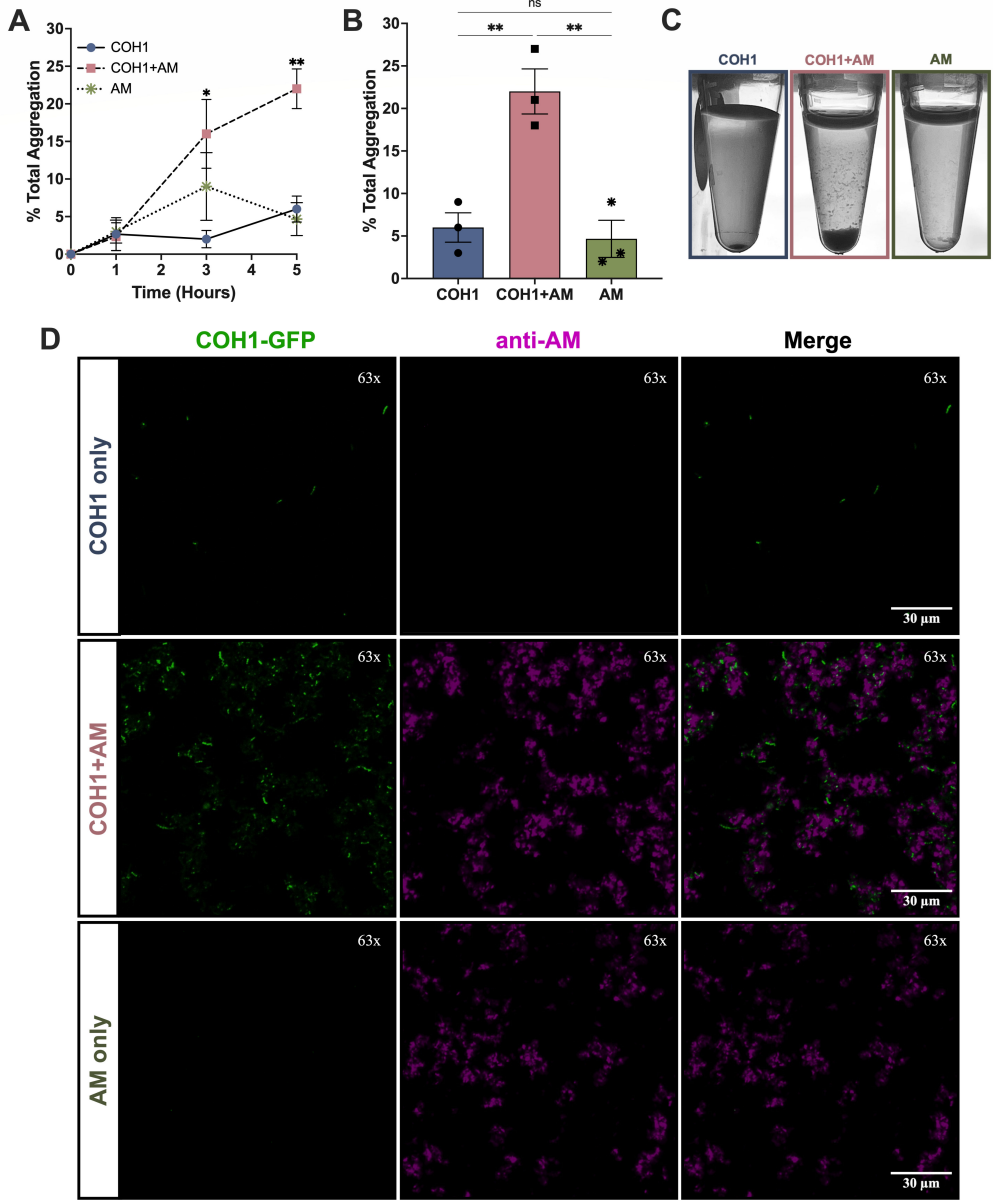
GBS and *A. muciniphila* co-aggregate *in vitro*. (**A**) OD_600_ was monitored at 0 h, 1 h, 3 h, and 5 h, and percent aggregation was calculated from initial OD_600_. (**B**) Bar graph and (**C**) image depict aggregation at 5 h. (**D**) Aggregates were examined by confocal fluorescence microscopy (representative images). Statistical analysis was determined by Student’s *t*-test. Data points represent the average of independent experiments. Error bars represent ±SEM. ns, > 0.05; *, *P* ≤ 0.05; **, *P* ≤ 0.01.

**Fig 3:**
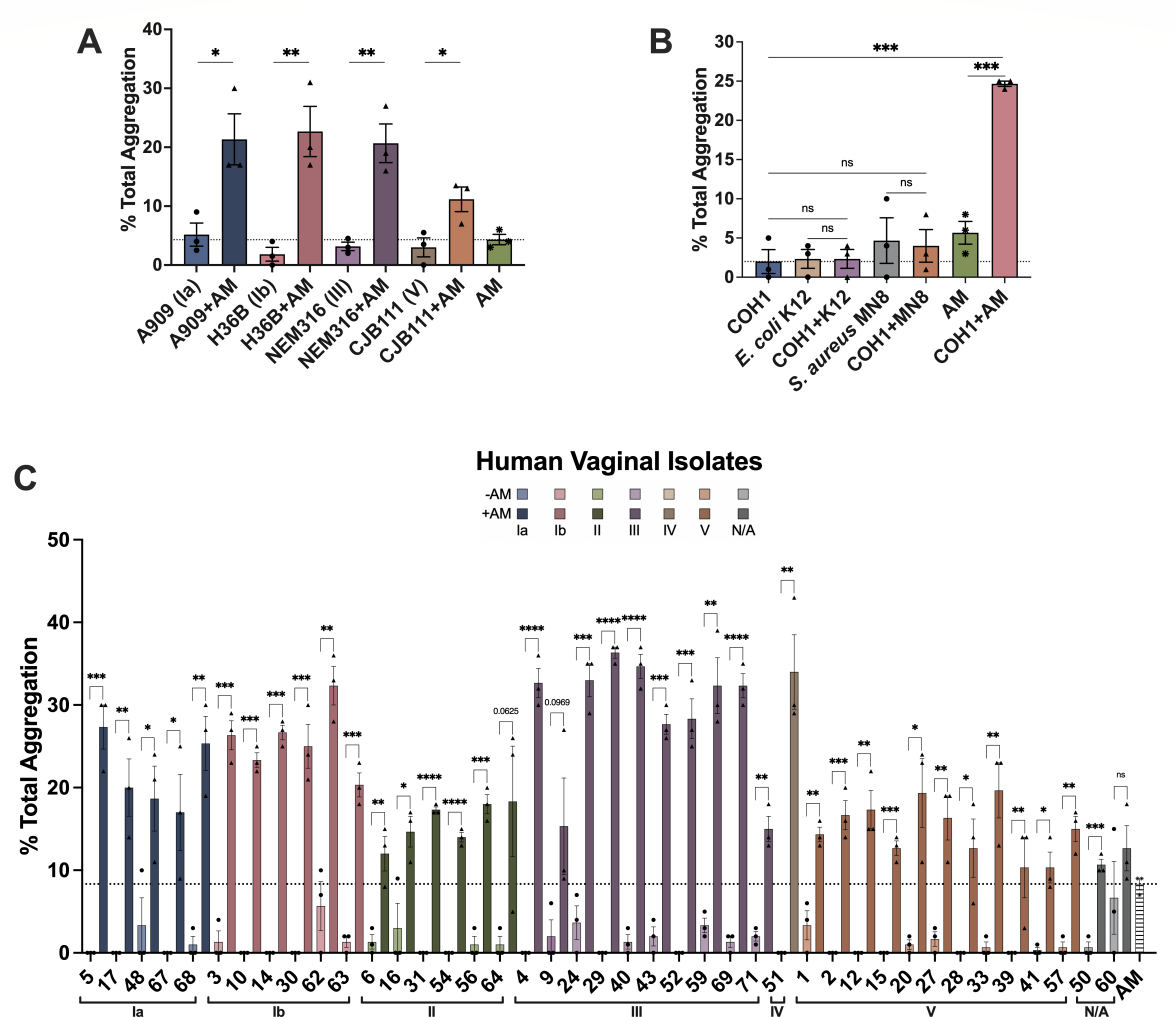
*A. muciniphila* interacts with various GBS capsular serotypes, sequencing types, and GBS vaginal isolates from pregnant individuals. (**A**) Aggregation of several GBS serotypes and sequencing types to *A. muciniphila* was examined. (**B**) Aggregation of GBS (COH1) ± *E. coli* K-12 (K12), *S. aureus* MN8 (MN8), and *A. muciniphila* was evaluated. (**C**) Aggregation of various GBS human vaginal isolates to *A. muciniphila* was assessed. Vaginal isolates are color-coded based on their capsular serotype with the lighter color as GBS only (*−A. muciniphila*) and the darker color as GBS + *A. muciniphila*. Capsular serotype is also denoted below graph. Bar graphs depict aggregation at 5 h. Statistical analysis was determined by Student’s *t*-test. Data points represent the (**A and B**) average of independent experiments or (**C**) technical replicates from two independent experiments. Error bars represent ±SEM. ns, > 0.05; *, *P* ≤ 0.05; **, *P* ≤ 0.01; ***, *P* ≤ 0.001; ****, *P* ≤ 0.0001.

**Fig 4:**
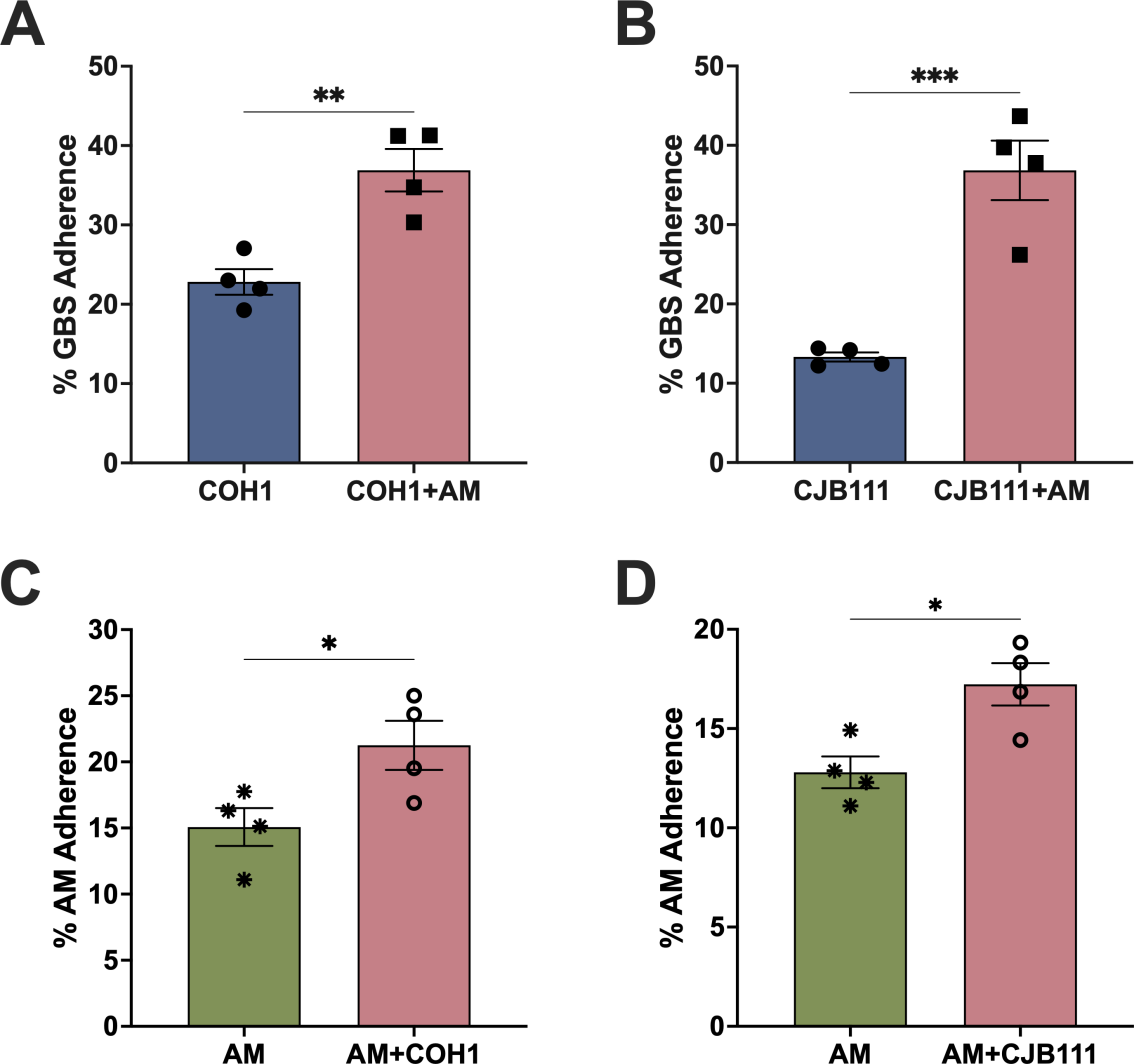
GBS and *A. muciniphila* reciprocally enhance adherence to human vaginal epithelial cells (hVECs). Human vaginal epithelial cells were grown to confluency in a 24-well tissue culture plate. GBS and *A. muciniphila* were grown overnight and standardized to an MOI of ~1, and co-infection was performed at a 1:1 ratio. Percent adherence for GBS strains (**A**) COH1 and (**B**) CJB111 ± *A. muciniphila* or for *A. muciniphila* (**C**) ± COH1 and (**D**) ± CJB111 was calculated. Statistical analysis was determined using a Student’s *t*-test. Data represent the average of independent experiments. Error bars represent ±SEM. *, *P* ≤ 0.05; **, *P* ≤ 0.01; ***, *P* ≤ 0.001.

While this work and our previously published results demonstrate that *A. muciniphila* is present in the vaginal tract and influences GBS abundance, whether these microbes are directly interacting is unknown. To begin investigating this, we incubated GBS strain COH1 (serotype III) and *A. muciniphila* in PBS and observed that the two bacteria aggregate significantly more together compared to their mono-cultures (Fig. 2A through C). These prominent aggregates can be further visualized by fluorescence microscopy using a GFP-expressing GBS strain and post-staining with an antibody against *A. muciniphila* (Fig. 2D). We examined *A. muciniphila* aggregation with other GBS clinical isolates of varying serotypes and found a significant increase in co-aggregation compared to GBS alone (Fig. 3A). Moreover, we investigated the capacity of GBS to aggregate in the presence of another Gram-negative bacterium, *Escherichia coli* K12, as well as a Gram-positive bacterium, *Staphylococcus aureus* MN8 (a menstrual toxic shock syndrome clinical isolate) (35). Importantly, we did not observe a significant increase in aggregation when GBS was co-incubated with either *E. coli* K12 or *S. aureus* MN8 (Fig. 3B). Lastly, we assessed aggregation of GBS strains isolated from the vaginal tract of pregnant women (36) with and without *A. muciniphila*. We saw significantly increased aggregation across all serotypes in the presence of *A. muciniphila* in all but three of the isolates (Fig. 3C). We next sought to examine GBS and *A. muciniphila* interactions on human vaginal epithelial cells (hVECs) using adherence assays (37). Here, we found that the presence of *A. muciniphila* significantly increased GBS adherence to hVECs by approximately 14.0% and 23.5% for strains COH1 and CJB111 (serotype V), respectively (Fig. 4A and B). Moreover, we also found that *A. muciniphila* adherence to hVECs was increased by the presence of GBS (Fig. 4C and D). These results reveal that *A. muciniphila* consistently promotes co-aggregation across diverse GBS isolates and that both bacteria enhance adherence to hVECs.

### RNA sequencing reveals unique GBS transcriptome changes during co-infection of hVECs

**Fig 5:**
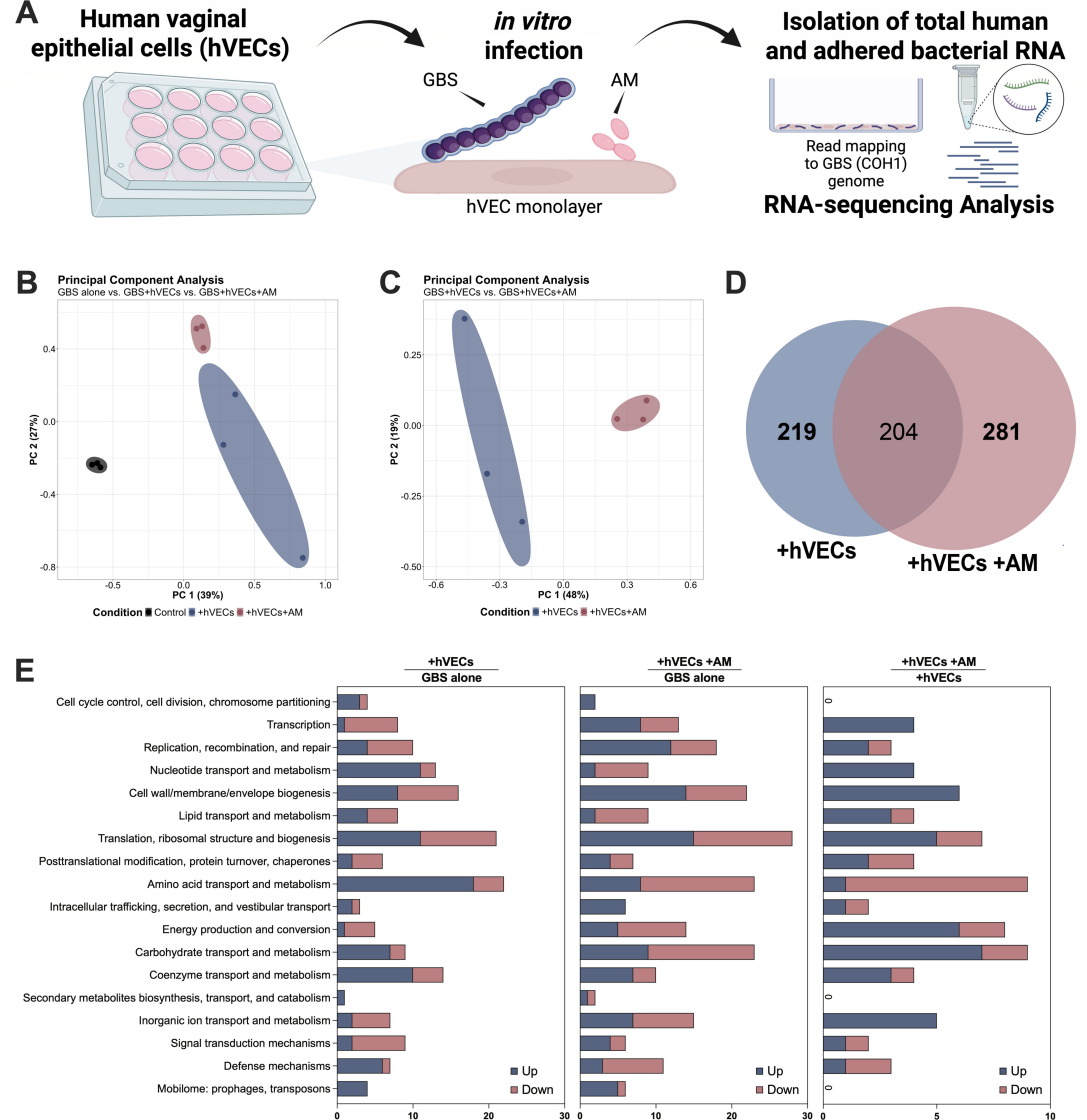
Impact of *A. muciniphila* on the GBS transcriptome. (**A**) Four-hour mono- and co-infections of human vaginal epithelial cells using GBS (COH1) and *A. muciniphila* at an MOI of ~100 for RNA-sequencing analysis. (**B and C**) PCA plot displays gene counts, and (**D**) Venn diagram compares transcriptomic changes for GBS under each condition. (**E**) GBS genes with an FDR *P*-value ≤0.05 and a fold change cutoff ≥|1.5| that were unique to each condition were sorted based on biological pathways. Data represent three biological replicates. Panel A was created using Biorender.com.

After observing the ability of GBS and *A. muciniphila* to co-aggregate and their increase attachment to hVECs, we next sought to determine the effect of *A. muciniphila* on GBS gene expression during infection of the vaginal epithelium. We performed RNA sequencing to examine global transcriptomic changes in GBS during mono-infection of hVECs (GBS + hVECs) and co-infection with *A. muciniphila* (GBS + AM + hVECs), alongside a GBS alone control (GBS grown in keratinocyte serum-free media [KSFM]) (Fig. 5A). Principal component analysis (PCA) revealed that each of the three conditions clustered separately, and we observed the greatest separation between the GBS medium control and the GBS adhered to hVECs (Fig. 5B). To specifically assess differences between infection conditions on hVECs, we repeated the PCA using only the mono-infection and co-infection samples, which formed distinct clusters and indicated transcriptional differences between these states (Fig. 5C). Using a fold change cutoff of ≥|1.5| and a false discovery rate (FDR)-adjusted *P*-value of ≤0.05 as parameters, we identified 219 and 281 genes that were uniquely altered during mono-infection and co-infection, respectively, and 204 genes that were shared (Fig. 5D; Table S1).

We next organized these uniquely altered genes by orthologous groups and broadly compared trends in gene expression between mono-infection and co-infection conditions (Fig. 5D). Here, we identified significant changes in regulation of carbohydrate transport and metabolism; intracellular trafficking, secretion, and vestibular transport; and energy production and conversion. During mono-infection, genes involved in amino acid transport and metabolism were predominantly upregulated and shifted to being predominantly downregulated during co-infection with *A. muciniphila* (Fig. 5E). Further, we observed a profound shift in cell wall/membrane/envelope biogenesis genes, where the majority of uniquely altered genes belonging to this functional group were equally regulated during mono-infection, as opposed to being predominantly upregulated during co-infection (Fig. S1A). This difference may reflect distinct environmental signals encountered during co-infection, such as direct interaction with *A. muciniphila* on hVECs, which could provide additional cues. Although the precise mechanism is not clear, co-infection appears to augment expression of these genes compared to hVECs alone. To further isolate the effect of *A. muciniphila*, we examined transcriptomic changes during co-infection with *A. muciniphila* by using the GBS transcriptome during mono-infection as a control. Using a fold change cutoff ≥|1.5| and a FDR-adjusted *P*-value of ≤0.05 as parameters, we identified 89 genes that were uniquely altered by *A. muciniphila* (Table S2). Here, we observed that genes involved in cell wall/membrane/envelope biogenesis were exclusively upregulated, genes in the carbohydrate transport and metabolism functional group were mostly upregulated, and, once more, genes involved in amino acid transport and metabolism were predominantly downregulated (Fig. 5E).

### GBS uniquely modulates cell envelope gene expression in the presence of *A. muciniphila*

**Fig 6:**
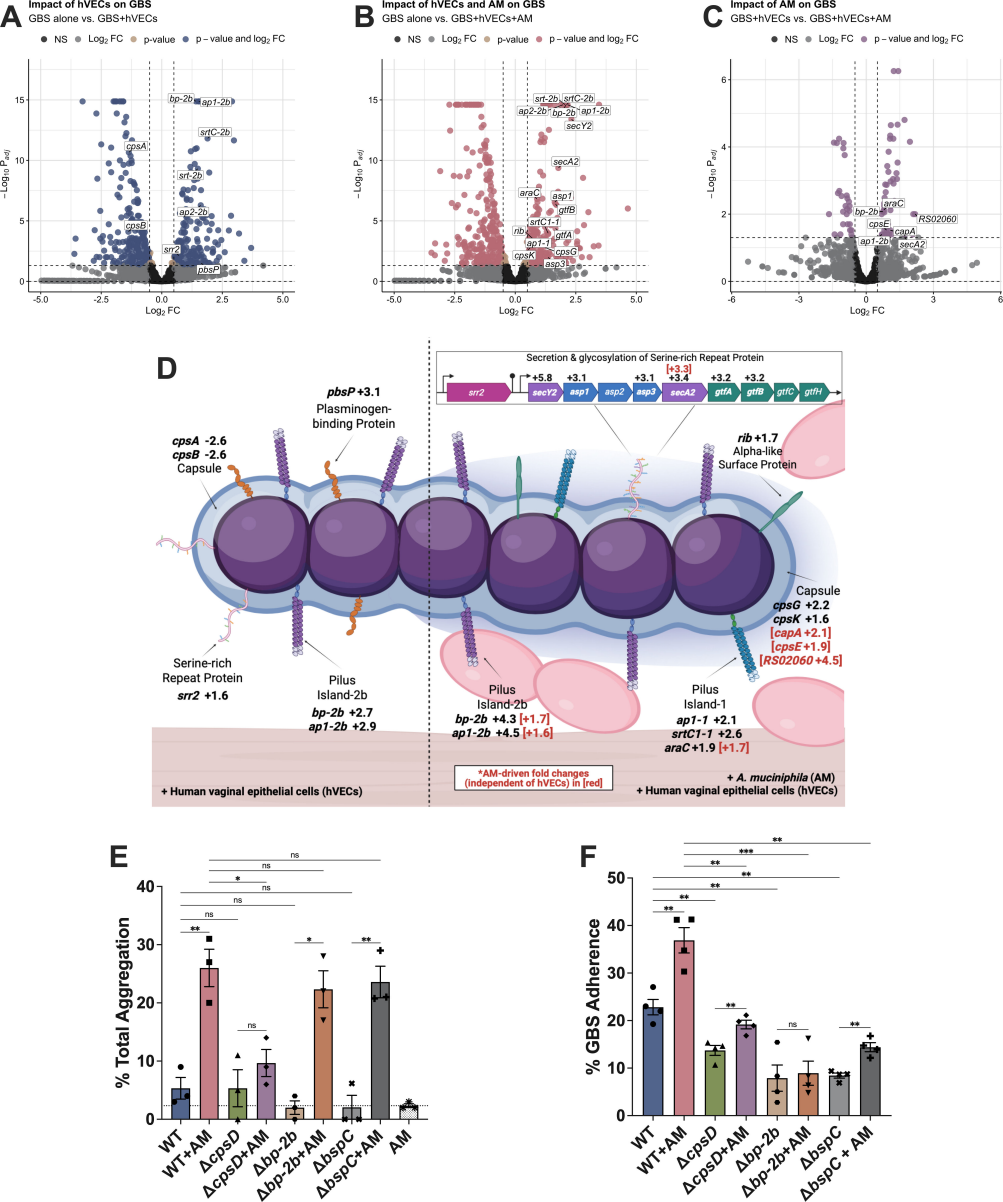
GBS uniquely modulates the cell envelope during mono- and co-infection. Volcano plots compare GBS gene expression (**A**) +hVECs – *A. muciniphila,* (**B**) +hVECs + *A. muciniphila*, or (**C**) +*A. muciniphila* (+hVECs transcriptome was used as a baseline to isolate the role of *A. muciniphila* on GBS gene expression). (**D**) Select GBS gene fold changes are shown and compared between both conditions; red text in brackets indicates fold changes corresponding to the RNAseq analysis from panel **C** which uses +hVECs as a control. Aggregation and adherence were evaluated for various GBS mutants. (**E**) The graph depicts aggregation at 5 h for GBS WT and Δ*cpsD* (Hy106), Δ*bp-2b*, Δ*bspC* mutants ± *A. muciniphila*. (**F**) The graph depicts percent adherence for GBS WT and Δ*cpsD* (Hy106), Δ*bp-2b*, Δ*bspC* mutants ± *A. muciniphila*. Statistical analysis was determined using a Student’s *t*-test (**E and F**) Data points represent the average of independent experiments. Error bars represent ±SEM. ns, > 0.05; *, *P* ≤ 0.05; **, *P* ≤ 0.01; ***, *P* ≤ 0.001. Panel **D** was created using Biorender.com.

We identified numerous genes, both unique to each condition and shared, and examined their expression. We decided to further examine the alterations in the expression of select genes involved in cell wall/membrane/envelope biogenesis (Fig. 6A through C). Here, we noted an interesting difference in regulation of GBS capsule biosynthesis genes, including *cpsA* and *cpsB,* which were significantly downregulated during mono-infection (Fig. 6A), while *cpsG* and *cpsK* were significantly upregulated during co-infection (Fig. 6B). Further, in our analysis of the GBS transcriptome during co-infection (GBS + AM + hVECs), using the mono-infection of hVECs (GBS + hVECs) as a control, we found upregulation of *capA*, *cpsE*, and *RS02060* (encoding a surface polysaccharide O-acyltransferase, an integral membrane enzyme) (Fig. 6C). We observed differences in expression of genes encoding pilus-island 2b (PI-2b) in all three analyses (Fig. 6A through C) and pilus-island 1 (PI-1) in both co-infection analyses (Fig. 6B and C). We also noted differential expression of genes encoding plasminogen-binding protein (PbsP) and serine-rich repeat protein 2 (Srr2) during mono-infection, whereas genes encoding alpha-like surface protein (Rib) and the Srr2 secretion and glycosylation system were differentially expressed during co-infection. Moreover, we found that the expression of genes involved in PI-2b synthesis, *bp-2b* and *ap1-2b*, was upregulated to a higher degree during co-infection, as compared to mono-infection (Fig. 6D). Conversely, the expression of genes involved in PI-1 synthesis, *araC*, *ap1-1,* and *srtC1-1* was only significantly upregulated during co-infection. Expression of PI-1 and PI-2b genes that were upregulated in our analysis using the GBS transcriptome during mono-infection as a control (*araC*, *bp-2b*, *ap1-2b*) was validated by RT-qPCR (Fig. 6C; Fig. S1B).

Another notable difference was observed in *srr2* expression during mono-infection, as this gene was not significantly altered during co-infection. However, in the presence of *A. muciniphila*, we observed the upregulation of numerous genes involved in the secretion and glycosylation of Srr2, including *secY2* and *secA2*, which encode subunits for the translocase in this secretion (Sec) system; *asp1* and *asp2*, which encode accessory proteins for this Sec system; and *gtfA* and *gtfB*, which encode a glycosyltransferase and glycosylation chaperone for this Sec system, respectively. Further, in our analysis using the GBS transcriptome during mono-infection as a control, we found upregulation of *secA2* (Fig. 6D). Importantly, we did not identify differences in notable regulators of virulence, including *saeR*, *saeS*, and *covS*, across our three analyses (Tables S1 and S2).

We sought to examine the role of specific GBS representative surface factors using GBS mutants in capsule (Δ*cpsD*), PI-2b (Δ*bp-2b*), and the group B streptococcal surface protein BspC (Δ*bspC*), which was used as an example of a gene that was not altered in our RNA-seq analyses but has previously been shown to contribute to GBS self-aggregation and adherence to hVECs (37). We observed that *A. muciniphila* significantly increased GBS aggregation with the Δ*bp-2b* and Δ*bspC* mutant strains similar to WT GBS but did not significantly increase aggregation with the Δ*cpsD* mutant, suggesting the importance of capsule for this interaction (Fig. 6E). We noted that *A. muciniphila* did not significantly increase adherence of the Δ*bp-2b* mutant to hVECs, whereas an increase in adherence was observed with the Δ*cpsD* and Δ*bspC* mutant strains (Fig. 6F). These findings suggest the importance of this pilus island-2b to the *A. muciniphila*-dependent increase in GBS hVEC attachment. Notably, we observed overall reduced adherence to hVECs with these GBS mutants, as has been published previously (36–38). Thus, we demonstrate that the increases in GBS aggregation and attachment to hVECs in the presence of *A. muciniphila* are dependent on GBS capsule and pili, respectively, and notably, *A. muciniphila* induces upregulation of both capsule and PI-2b.

### GBS vaginal colonization is significantly reduced during continual intravaginal treatment with *A. muciniphila*

**Fig 7:**
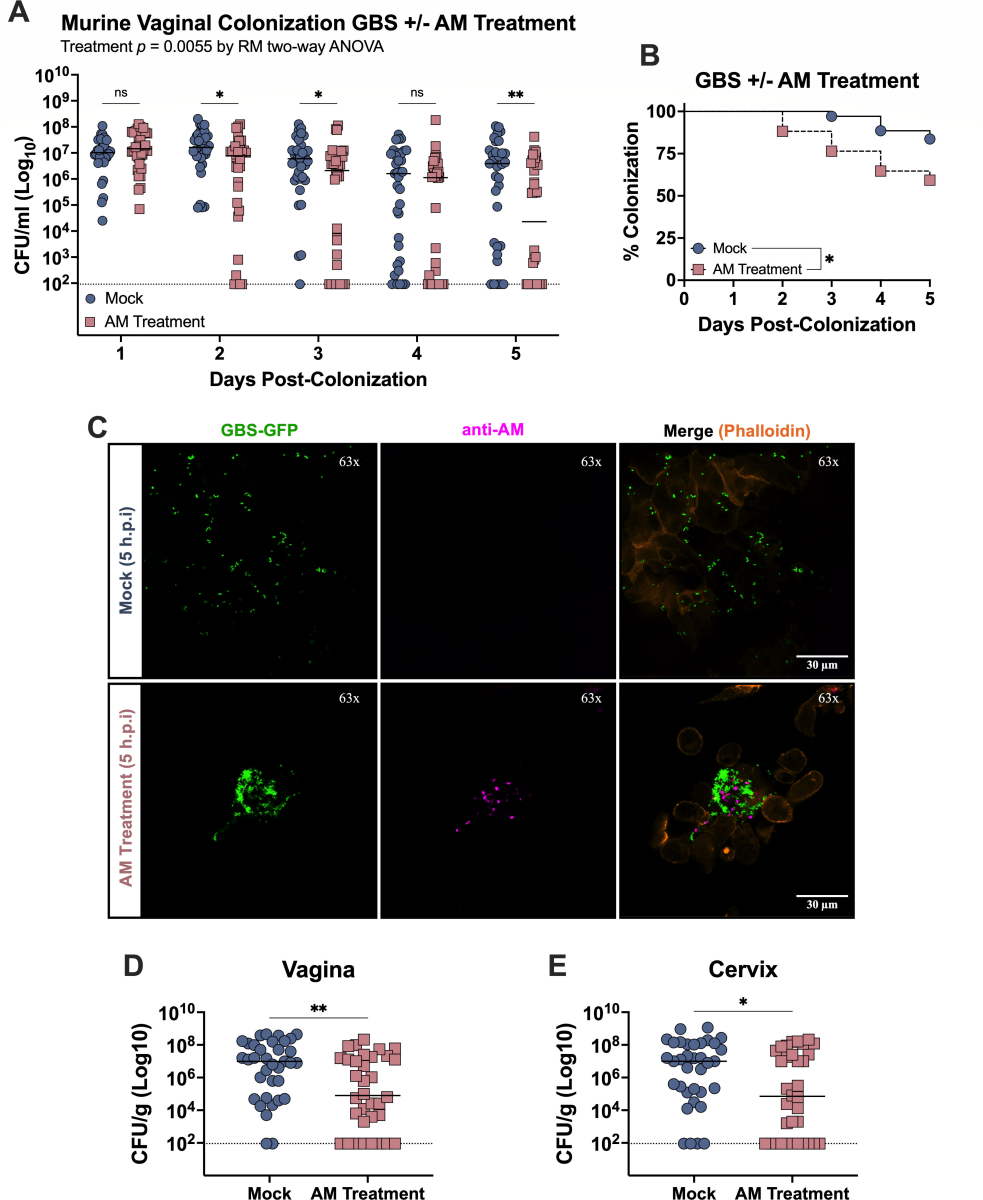
*A. muciniphila* treatment significantly reduces GBS colonization and invasion of the murine cervicovaginal mucosa (**A**) recovered GBS CFU from daily lavage of the vaginal lumen and (**B**) corresponding colonization curve. (**C**) Fluorescence microscopy of pooled vaginal lavage 5 h post*-A. muciniphila* treatment on day 1 (representative images). Recovered GBS CFU counts from (**D**) vaginal and (**E**) cervical tissues at day 5. Murine data represent three independent experiments, *n* = 10–15 mice per group, per experiment. Microscopy represents three biological replicates. Solid lines represent the median. Statistical analysis was performed by (**A**) RM two-way ANOVA with Holm-Šídák’s multiple comparisons test, (**C**) log-rank (Mantel-Cox) test, or (**D and E**) Mann-Whitney *U* test. ns, > 0.05; *, *P* ≤ 0.05; **, *P* ≤ 0.01.

Our results provide further evidence that *A. muciniphila* and GBS co-occur in the human vaginal tract and suggest that their co-aggregation and enhanced bacterial adherence to vaginal epithelium may provide a mechanism for increased GBS colonization in humans and in our previously published mouse model (22). Due to the recent interest in using *A. muciniphila* in oral supplements for probiotic therapy (25–27), we sought to examine the effect of daily *A. muciniphila* vaginal treatment on GBS persistence. Using our well-established murine model of GBS vaginal colonization (39), CD-1 mice were synchronized to estrus using 17β-estradiol and intravaginally inoculated with GBS or with GBS and *A. muciniphila*. Mice were dosed every day with *A. muciniphila* (treatment) or PBS (mock), and the vaginal lumen was lavaged daily to enumerate GBS burden until 5 days post-colonization when tissues were harvested. We found that daily treatment with *A. muciniphila* significantly reduced GBS burdens compared to the mock treatment group and that the decrease is most discernible on day 5 post-colonization with a 2.2-log reduction in median CFU/mL observed in the *A. muciniphila* treatment group (Fig. 7A). This corresponded to a significant decrease in GBS colonization over time (Fig. 7B). Examination of vaginal lavage by fluorescence microscopy showed that GBS appeared to be more aggregated in the presence of *A. muciniphila* (Fig. 7C). Importantly, this decrease in GBS burden with *A. muciniphila* treatment was also observed in vaginal and cervical tissues collected on day 5 post-colonization (Fig. 7D and E). These data suggest that daily supplementation with exogenous *A. muciniphila* over time could be beneficial in reducing GBS colonization.

## DISCUSSION

To date, *A. muciniphila* has been predominantly studied in the context of GI health, specifically for its immunomodulatory role and beneficial effect to human health as a probiotic supplement (25–27). However, the global effect of *A. muciniphila* in other niches and its relationship with GBS remain largely understudied. Work published in 2018 first depicted a relationship between GBS and *Akkermansia* spp., where it was found that an attenuated GBS strain increased the relative abundance of *Akkermansia* spp. from 1% to 28% in the gut of Nile tilapia (40). Our lab recently described for the first time the association between *Akkermansia* spp. and GBS in the murine vaginal tract and the co-occurrence of GBS and *A. muciniphila* in pregnant women (22). Here, we present additional evidence that GBS and *A. muciniphila* co-occur in the human FGT and demonstrate that *A. muciniphila* is present in 12–72% of cervicovaginal samples across independent metagenomic studies. Cross-talk between the gut and FGT microbiota has been shown to influence host physiology, and members of the FGT microbiota can be traced back to the rectum, which serves as a microbial reservoir (41). Notably, research has shown that the same bacterial species have been identified in paired rectal and vaginal samples from pregnant individuals, with the vast majority of these bacteria demonstrating identical genotypes (42). As such, it is possible that *A. muciniphila* seeds the vagina in individuals who are colonized in the GI tract.

Independent of our research, recent findings have begun to implicate *Akkermansia* in maternal health and pregnancy outcomes. For example, depletion of *Akkermansia* spp. in the GI tract of pregnant individuals was found to be associated with preeclampsia (PE), a pregnancy-specific multisystem disorder that affects 2–8% of pregnancies and is a leading cause of maternal morbidity (43). Importantly, it was shown that oral treatment with pasteurized *A. muciniphila* significantly promoted fetal growth and improved placental pathology in a murine model of PE (43). Moreover, the relative abundance of *Akkermansia* spp. was shown to be negatively associated with blood glucose levels in individuals with gestational diabetes mellitus (GDM), and oral treatment with either live or pasteurized *A. muciniphila* significantly improved adverse effects of GDM in various studies (44–46). Interestingly, GDM has been associated with an increased risk for maternal GBS colonization, as well as worse neonatal outcomes associated with GBS infection (47). Together, these data suggest beneficial potential for *A. muciniphila* in maternal-fetal health. In future work, we aim to investigate how *A. muciniphila* influences long-term GBS persistence and ascending infection during pregnancy.

RNA sequencing analysis identified important GBS surface factors that were modulated in the presence of hVECs and *A. muciniphila*. For example, PI-2b has previously been shown to be necessary for attachment to cells of the FGT, binding to host mucins, and vaginal colonization *in vivo* (48). We found that PI-2b expression is upregulated to a higher degree during co-infection with *A. muciniphila* compared to mono-infection. It is possible that *A. muciniphila* directly upregulates PI-2b expression; however, it is also possible that *A. muciniphila* modifies the host environment and thereby indirectly results in the upregulation of PI-2b expression. Interestingly, interaction with *A. muciniphila* appears to be dependent on some, but not all, of the surface factors that we found to be differentially expressed, as PI-2b is required for the *A. muciniphila-*mediated increase in adherence, but not for direct co-aggregation with *A. muciniphila*. These data highlight the complex mechanisms that may be involved in interbacterial aggregation compared to those involved in attachment to host cells.

The 10 known GBS capsular serotypes have distinct molecular compositions, where the arrangement and structure of the base monosaccharides (glucose, galactose, and N-acetylglucosamine) vary enough to be antigenically distinct. However, all known GBS serotypes contain terminally linked sialic acid (Sia) (49, 50). We identified numerous GBS genes involved in capsule production to be significantly upregulated during co-infection of hVECs, including a glycosyltransferase involved in adding glucose to the galactose base sugar (*cpsG*) (49) and a sialyltransferase that adds the ⍺2,3-linked terminal Sia to the repeating sugar unit (*cpsK*) (51). Interestingly, the human GI tract is lined by mucus comprised of heavily O-glycosylated secreted and membrane-anchored mucins, and an increasing gradient of sialylation of *O*-glycans in mucin is observed in the distal colon of humans, a niche that *A. muciniphila* inhabits (52–54). Recent work has shown that the O-glycans on these mucins serve as an attachment point and nutrient source for *A. muciniphila* and that endogenous sialidase activity of *A. muciniphila* enhances binding to sialylated mucins by removing the Sia. The sialylated capsule protects GBS from complement deposition and phagocytosis, enhances biofilm formation, inhibits the binding of antimicrobial peptides, and alters adherence to cells and mucins (49). Further investigation is required to determine if *A. muciniphila* cleaves the terminal Sia on GBS capsule, which would likely impact GBS susceptibility to phagocytic clearance; this remains an avenue of our future studies.

In our previous studies, Burcham et al. first identified *Akkermansia* spp. in the vaginal microbiome and demonstrated an association to GBS vaginal colonization (22). Using a murine model of GBS vaginal colonization, this previous work demonstrated that intravaginal pre-treatment with *A. muciniphila* or singular co-inoculation of *A. muciniphila* and GBS resulted in enhanced GBS persistence in the vaginal lumen. Our analysis of additional human data sets provides more evidence that *A. muciniphila* and GBS co-occur in the human vaginal tract across diverse groups. Additionally, our current findings that *A. muciniphila* and GBS co-aggregate and increase bacterial adherence to vaginal epithelium suggest a mechanism for the enhancement of GBS colonization in humans and in mice. Because companies are currently selling oral probiotic supplements containing *A*. *muciniphila*, we decided to examine the impact of continual *A. muciniphila* vaginal inoculation on GBS vaginal persistence in our mouse model. Interestingly, we observed that daily intravaginal treatment with *A. muciniphila* significantly reduced GBS bacterial loads in the vaginal lumen and in the cervicovaginal mucosa. This could be due to GBS-*A. muciniphila* co-aggregation, which we observed in the vaginal lumen *in vivo*, but additional studies are required to determine when these interactions are beneficial to the host by limiting GBS access to FGT tissues versus promoting GBS persistence.

In summary, we report for the first time interactions between GBS and *A. muciniphila* and demonstrate that *A. muciniphila* has a significant impact on GBS aggregation, host cell interaction, and gene expression during vaginal colonization. Further studies are required to elucidate the mechanisms by which *A. muciniphila* modulates GBS vaginal colonization, including potential modification of GBS capsule composition, as well as modulation of the microbiome and host mucosal immunity. In addition, our studies provide the first evidence for the beneficial potential of *A. muciniphila* in the vagina to limit an opportunistic pathogen like GBS. These observations provide a platform for continued studies of *A. muciniphila* in the FGT, which remains a focus of ongoing work in our laboratory.

## MATERIALS AND METHODS

### Bacterial strains and growth conditions

*Streptococcus agalactiae* (GBS) isolates A909 (serotype Ia), H36B (serotype Ib), COH1 (serotype III), NEM316 (serotype III), CJB111 (serotype V), and 41 vaginal isolates from pregnant women were cultured statically in Todd-Hewitt (TH; Research Products International, RPI) broth at 37°C unless stated otherwise. When necessary, GBS was grown on TH agar. *Akkermansia muciniphila* (Muc^T^; ATCC BAA-835) was cultured statically in pre-reduced brain-heart infusion (BHI; RPI) broth supplemented with 0.1% porcine gastric mucin (PGM; Sigma-Aldrich) at 37°C unless stated otherwise. When necessary, *A. muciniphila* was grown on BHI agar with 0.1% PGM and 5 µg/mL erythromycin to select against GBS. *A. muciniphila* was cultured anaerobically in a Coy Laboratory Products Type A, vinyl anaerobic chamber using an atmospheric gas mix of 85% N_2_/10% CO_2_/5% H_2_ unless stated otherwise. *Escherichia coli* K-12 was grown shaking in lysogeny broth (RPI) at 37°C and *Staphylococcus aureus* was grown shaking in tryptic soy broth (BD Difco) at 37°C.

### Tissue culture conditions

Human vaginal epithelial (VK2/E6E7; ATCC Cat# CRL-2616) cell line (55) was obtained from the American Type Culture Collection and was maintained in KSFM (Gibco) supplemented with 0.5 ng/mL human recombinant epidermal growth factor and 0.05 mg/mL bovine pituitary extract at 37°C in 5% CO_2_.

### Animal studies

Seven-week-old female CD-1 mice were purchased from Charles River Laboratories. Mice were housed in appropriate ABSL facilities at the University of Colorado Anschutz Medical Campus (CU-AMC) and allowed to acclimate for at least one week prior to experimentation.

### Metagenomic data analysis

Four publicly available vaginal metagenomics data sets from pregnant and non-pregnant individuals were analyzed for trends between GBS and *A. muciniphila*. Normalized read counts with assigned taxa were downloaded from Baud et al. (31). Raw reads were downloaded from Tortelli et al. (32), France et al. (33), and Jung et al. (34) and processed using BBduk (BBmap v39.52), Hostile (v2.0.2), Kraken2 (v2.1.5), and Bracken (v2.9) for trimming, host filtering, read classification, and taxonomic abundance quantification, respectively. Samples were binned as positive for a microbe if they contained at least one normalized read assigned to the associated taxon. The number of samples positive for *S. agalactiae*, *A. muciniphila*, both, and neither was used to identify statistical correlation. Normalized read counts for *S. agalactiae* in the *A. muciniphila* positive or negative samples were also assessed.

### Bacterial aggregation assays

GBS and *A. muciniphila* were grown overnight, washed, and standardized to an OD_600_ of 1.0 in phosphate-buffered saline (PBS). Mono-cultures were examined at a 1:1 ratio (bacteria to PBS), and co-cultures were examined at a 1:1 ratio (GBS to *A. muciniphila*, *E. coli*, or *S. aureus*) in a total volume of 1 mL at room temperature (RT). Samples were mixed vigorously, and 10 µL of sample was taken from the top of each sample immediately and diluted in 90 µL of PBS to measure optical density using a plate reader. OD_600_ was examined at hours 1, 3, and 5, and percent aggregation was calculated based on OD_600_ at hour 0.

### Bacterial adherence assays

Bacterial adherence assays were performed to determine the total number of cell-surface adhered bacteria as previously described (38). GBS was grown overnight and subcultured (1:10) in fresh media to mid-logarithmic phase and standardized to an OD_600_ of 0.4 (CFU ~1 × 10^8^). *A. muciniphila* was grown overnight and standardized to an OD_600_ of 0.4 (CFU ~4 × 10^8^). hVECs were infected at a multiplicity of infection (MOI) of ~1 and incubated for 30 min at 37°C in 5% CO_2_. Co-infection was performed at a 1:1 ratio of GBS and *A. muciniphila*. No evidence of cell toxicity was observed by microscopy following the 30-minute incubation. Then, hVECs were gently washed four times with sterile PBS, released from the well with 100 µL of 0.25% trypsin-EDTA (Thermo Fisher Scientific), and lysed with 400 µL of 0.025% Triton X-100 (Thermo Fisher Scientific) for a total final volume of 500 µL. Cell lysates were serially diluted and plated to enumerate CFU alongside the bacterial input. Percent adherence was calculated based on input.

### Confocal fluorescence microscopy

Samples were examined by fluorescence microscopy to determine spatial distribution of bacterial cells. Bacterial aggregates or murine vaginal lavage samples were fixed in 4% paraformaldehyde for 30 min at RT. Samples were washed three times and stored in 1% bovine serum albumin in PBS until staining. For microscopy, GFP-expressing COH1 and CJB111 strains were used to image GBS in bacterial aggregates and murine vaginal lavage, respectively. For staining, rabbit anti-*A*. *muciniphila* polyclonal primary antibody (Sigma-Aldrich) paired with goat anti-rabbit IgG Alexa Fluor 633 secondary antibody (Sigma-Aldrich) was used to detect *A. muciniphila*, and phalloidin-iFluor 555 (Abcam) was used to visualize F-actin. Samples were imaged using an LSM 780 inverted laser scanning confocal microscope (Zeiss) with the 63×/NA 1.40 oil immersion objective lens using the 488 nm, 561 nm, and 633 nm continuous wave lasers. Image acquisition was performed using Zen Black software and processed using Fiji software.

### Bacterial infection of human vaginal epithelial cells for transcriptomics analysis

GBS was grown in biological triplicate overnight and subcultured (1:10) in fresh media to mid-logarithmic phase and standardized to an OD_600_ of 0.4 (CFU ~1 × 10^8^). *A. muciniphila* was grown in biological triplicate overnight and standardized to an OD_600_ of 0.4 (CFU ~4 × 10^8^). hVEC monolayers were infected at a multiplicity of infection (MOI) of ~100; plates were centrifuged at 200 × *g* for 5 min and incubated for 4 h at 37°C in 5% CO_2_. Co-infection was performed at a 1:1 ratio of GBS and *A. muciniphila* (Total bacterial MOI = 200). Input was serially diluted and plated to confirm appropriate MOI. Immediately following infection, supernatant was removed. This was done to retain only bacteria actively attached to hVECs. Following, 1 mL of RLT lysis buffer (Qiagen) supplemented with 0.1% β-mercaptoethanol (Sigma-Aldrich) was added to each well and pipetted up and down vigorously, with light scraping to ensure hVEC detachment from well, and samples were frozen at −80°C.

### RNA isolation, library preparation, and RNA sequencing

Frozen samples were thawed on ice and transferred to 2.0 mL conical screw cap tubes with 0.1 mm zirconia beads. Samples were placed into a Mini BeadBeater (BioSpec) and homogenized two times for 40 s, followed by 1 min of ice in between each bead-beating step. Samples were centrifuged for 30 s at 17,000 × *g*, and supernatant was collected and placed in a new tube containing 70% molecular grade ethanol. Total RNA was prepared using an RNeasy Kit (Qiagen) as previously described (56). Once isolated, DNA was removed using a Turbo DNA-free Kit (Invitrogen). RNA concentrations were measured using a NanoDrop spectrophotometer to ensure a concentration of ≥50 ng/µL in 25 µL. RNA samples were sent overnight to SeqCenter (Pittsburg, PA) on dry ice. Samples were treated with Invitrogen DNase (RNase free), and library preparation was performed using the Stranded Total RNA Prep Ligation with Ribo-Zero Plus Kit (Illumina) and 10 bp unique dual indices (UDI). Sequencing was performed using a NovaSeq X Plus, which produced paired-end 151 bp reads. Demultiplexing, quality control, and adapter trimming were performed with bcl-convert (v4.1.5). Sequencing statistics were included with raw reads. Sequencing depth was high across all samples.

RNA-sequencing analysis was performed as previously described (56). Raw data (.fastq) files were uploaded to the CLC Genomics Workbench (Qiagen; v21.0.5) for analysis using default settings (mismatch cost: 2; insertion and deletion cost: 3; length and similarity fraction: 0.8). Paired reads corresponding to GBS rRNA, *A. muciniphila* rRNA, and human rRNA were removed by aligning to known rRNA sequences and discarded. The remaining unmapped paired reads (−rRNA) were aligned to the *S. agalactiae* COH1 reference genome (GenBank accession number: NZ_HG939456.1), and expression values were calculated using the RNA-seq analysis function with default mapping and expression parameters. Experimental comparisons were carried out following quantile normalization using the RNA-seq experimental fold change feature. Expression values calculated for each gene are shown as normalized reads per kilobase per million values (RPKM). Differentially expressed genes with an FDR adjusted *P*-value ≤0.05 (determined by CLC Genomics Workbench) and a fold change ≥|1.5| were considered for further analysis. Transcripts were annotated using GenBank (accession: NZ_HG939456.1), and COGs were assigned to differentially expressed genes. PCA plots and volcano plots were generated using R Studio (v 4.3.1.) with packages ggplot2, ggforce, edgeR, EnhancedVolcano. Conditions with GBS alone had ≥2.81 × 10⁷ read pairs, GBS with hVECs had ≥1.16 × 10⁸ read pairs, and the three-organism condition had ≥1.33 × 10⁸ read pairs. Full per-sample read counts and mapping statistics are provided in Table S3.

### RT-qPCR analysis

RNA was isolated and prepared as described above from two independent infections performed in biological triplicate. cDNA was made using the Quanta qScript cDNA Synthesis Kit following manufacturer’s guidelines. qPCR was performed using QuantaBio PerfeCTa qPCR FastMix and BioRad CFX96 Real-Time System. qPCR plates were loaded in technical duplicate.

### Murine vaginal colonization

One day prior to colonization, female CD-1 mice were injected intraperitoneally with 0.5 mg β-estradiol in 100 μL sesame oil to synchronize their estrous cycles and were vaginally lavaged with 100 μL of sterile PBS by pipetting 50 μL of sterile PBS (approximately eight times up and down) and repeating once more with 50 μL of fresh sterile PBS. Undiluted vaginal lavage was spot-plated on CHROMagar StrepB selective chromogenic media and incubated overnight at 37°C to confirm the absence of GBS for the study.

Following synchronization, mice were intravaginally inoculated directly with ~10^7^ CFU of mid-log phase GBS (CJB111) in 10 μL per mouse. For co-inoculation, mice were intravaginally inoculated directly with ~10^7^ CFU of mid-log phase GBS and ~10^7^ CFU of stationary phase *A. muciniphila* in 10 μL per mouse (5 μL per strain). Following inoculation, mice were vaginally lavaged daily as described above. Vaginal lavage was vortexed briefly, serially diluted 10^−1^ through 10^−4^, track-plated on CHROMagar StrepB, and incubated overnight at 37°C. Undiluted samples were spot-plated for every day post-inoculation. Vaginal lavaging and administration of PBS (mock) or ~10^7^ CFU *A*. *muciniphila* (treatment) were performed daily through experimental end-point.

Lavage data represent three independent experiments with *n* = 10–15 mice per group, per experiment. In an independent experiment, mice were vaginally lavaged on day 1 for confocal microscopy. This was performed following 5 h post-inoculation (hpi) with either PBS (mock) or *A. muciniphila* (treatment). Microscopy data represent pooled replicates from one independent experiment with *n* = 3 mice per group.

On day 5 of colonization, the reproductive tract of mice was harvested to enumerate GBS burden. Mice were humanely euthanized by primary CO_2_ and secondary cervical dislocation. Immediately afterward, vaginal lavage was collected as described above. Then, the vagina and cervix were dissected and placed into separate 2 mL screw-capped tubes containing ~1 cm of 1 mm zirconia beads and 500 μL sterile PBS. Tubes were weighed before and after adding tissues to calculate tissue weight. Homogenization was performed by bead beating for 1 min at maximum speed, followed by resting on ice for 1 min, for a total of two repetitions. Tissue homogenates were vortexed briefly, serially diluted 10^−1^ through 10^−4^, track-plated on CHROMagar StrepB, and incubated overnight at 37°C. Undiluted samples were spot-plated for every tissue. Tissue data are from three independent experiments, *n* = 10–15 mice per group, per experiment.

### Quantification and statistical analysis

All statistical analyses were performed using Prism software for MacOS (GraphPad Software; v10), and statistical significance was accepted at *P*-values of ≤0.05. RNA-seq analysis and statistics were performed using CLC Workbench. Differentially expressed genes with an FDR *P*-value ≤0.05 were considered significant in RNA-sequencing analyses, and differences with a *P*-value ≤0.05 were considered significant for all other experiments. The number of animals or sample size, bars, *P*-values, and specific statistical tests are specified in the corresponding figure legends.

## Data Availability

Raw data are available through the NCBI GEO repository (accession: GSE306314).

## SUPPLEMENTAL MATERIAL

Figure S1 mbio.02868-25-s0001.tiff Heat map and RT-qPCR.

Supplemental Material Legends mbio.02868-25-s0002.docx Figure S1 and Table S1-S3 legends.

Table S1 mbio.02868-25-s0003.xlsx GBS vs GBS + hVECs and GBS vs GBS + hVECs + AM.

Table S2 mbio.02868-25-s0004.xlsx GBS + hVECs vs GBS + hVECs + AM.

Table S3 mbio.02868-25-s0005.xlsx RNAseq statistics.

ASM does not own the copyrights to Supplemental Material that may be linked to, or accessed through, an article. The authors have granted ASM a non-exclusive, world-wide license to publish the Supplemental Material files. Please contact the corresponding author directly for reuse.

## References

[1] Russell NJ, Seale AC, O’Driscoll M, O’Sullivan C, Bianchi-Jassir F, Gonzalez-Guarin J, Lawn JE, Baker CJ, Bartlett L, Cutland C, Gravett MG, Heath PT, Le Doare K, Madhi SA, Rubens CE, Schrag S, Sobanjo-Ter Meulen A, Vekemans J, Saha SK, Ip M, GBS Maternal Colonization Investigator Group. 2017. Maternal colonization with group B Streptococcus and serotype distribution worldwide: systematic review and meta-analyses. Clin Infect Dis 65:S100–S111. doi:10.1093/cid/cix658 29117327 PMC5848259

[2] Nan C, Dangor Z, Cutland CL, Edwards MS, Madhi SA, Cunnington MC. 2015. Maternal group B Streptococcus-related stillbirth: a systematic review. BJOG 122:1437–1445. doi:10.1111/1471-0528.13527 26177561

[3] Vornhagen J, Armistead B, Santana-Ufret V, Gendrin C, Merillat S, Coleman M, Quach P, Boldenow E, Alishetti V, Leonhard-Melief C, Ngo LY, Whidbey C, Doran KS, Curtis C, Waldorf KMA, Nance E, Rajagopal L. 2018. Group B Streptococcus exploits vaginal epithelial exfoliation for ascending infection. J Clin Invest 128:1985–1999. doi:10.1172/JCI97043 29629904 PMC5919824

[4] Pass MA, Gray BM, Dillon HC Jr. 1982. Puerperal and perinatal infections with group B streptococci. Am J Obstet Gynecol 143:147–152. doi:10.1016/0002-9378(82)90644-5 7044126

[5] Vornhagen J, Adams Waldorf KM, Rajagopal L. 2017. Perinatal group B streptococcal infections: virulence factors, immunity, and prevention strategies. Trends Microbiol 25:919–931. doi:10.1016/j.tim.2017.05.013 28633864 PMC5650539

[6] Zhu Y, Lin XZ. 2021. Updates in prevention policies of early-onset group B streptococcal infection in newborns. Pediatr Neonatol 62:465–475. doi:10.1016/j.pedneo.2021.05.007 34099416

[7] Simonsen KA, Anderson-Berry AL, Delair SF, Davies HD. 2014. Early-onset neonatal sepsis. Clin Microbiol Rev 27:21–47. doi:10.1128/CMR.00031-13 24396135 PMC3910904

[8] Doran KS, Nizet V. 2004. Molecular pathogenesis of neonatal group B streptococcal infection: no longer in its infancy. Mol Microbiol 54:23–31. doi:10.1111/j.1365-2958.2004.04266.x 15458402

[9] Uchiyama S, Sun J, Fukahori K, Ando N, Wu M, Schwarz F, Siddiqui SS, Varki A, Marth JD, Nizet V. 2019. Dual actions of group B Streptococcus capsular sialic acid provide resistance to platelet-mediated antimicrobial killing. Proc Natl Acad Sci USA 116:7465–7470. doi:10.1073/pnas.1815572116 30910970 PMC6462088

[10] van Sorge NM, Quach D, Gurney MA, Sullam PM, Nizet V, Doran KS. 2009. The group B streptococcal serine-rich repeat 1 glycoprotein mediates penetration of the blood-brain barrier. J Infect Dis 199:1479–1487. doi:10.1086/598217 19392623 PMC2674616

[11] Rosini R, Rinaudo CD, Soriani M, Lauer P, Mora M, Maione D, Taddei A, Santi I, Ghezzo C, Brettoni C, Buccato S, Margarit I, Grandi G, Telford JL. 2006. Identification of novel genomic islands coding for antigenic pilus-like structures in Streptococcus agalactiae. Mol Microbiol 61:126–141. doi:10.1111/j.1365-2958.2006.05225.x 16824100

[12] Toniolo C, Balducci E, Romano MR, Proietti D, Ferlenghi I, Grandi G, Berti F, Ros IMY, Janulczyk R. 2015. Streptococcus agalactiae capsule polymer length and attachment is determined by the proteins CpsABCD. J Biol Chem 290:9521–9532. doi:10.1074/jbc.M114.631499 25666613 PMC4392257

[13] Lazzarin M, Mu R, Fabbrini M, Ghezzo C, Rinaudo CD, Doran KS, Margarit I. 2017. Contribution of pilus type 2b to invasive disease caused by a Streptococcus agalactiae ST-17 strain. BMC Microbiol 17:148. doi:10.1186/s12866-017-1057-8 28673237 PMC5496222

[14] Chang YC, Olson J, Beasley FC, Tung C, Zhang J, Crocker PR, Varki A, Nizet V. 2014. Group B Streptococcus engages an inhibitory siglec through sialic acid mimicry to blunt innate immune and inflammatory responses in vivo. PLoS Pathog 10:e1003846. doi:10.1371/journal.ppat.1003846 24391502 PMC3879367

[15] Tazi A, Disson O, Bellais S, Bouaboud A, Dmytruk N, Dramsi S, Mistou MY, Khun H, Mechler C, Tardieux I, Trieu-Cuot P, Lecuit M, Poyart C. 2010. The surface protein HvgA mediates group B Streptococcus hypervirulence and meningeal tropism in neonates. J Exp Med 207:2313–2322. doi:10.1084/jem.20092594 20956545 PMC2964583

[16] Tavares T, Pinho L, Bonifácio Andrade E. 2022. Group B streptococcal neonatal meningitis. Clin Microbiol Rev 35:e00079-21. doi:10.1128/cmr.00079-21 35170986 PMC8849199

[17] Patras KA, Nizet V. 2018. Group B streptococcal maternal colonization and neonatal disease: molecular mechanisms and preventative approaches. Front Pediatr 6:27. doi:10.3389/fped.2018.00027 29520354 PMC5827363

[18] Moncla BJ, Chappell CA, Debo BM, Meyn LA. 2016. The effects of hormones and vaginal microflora on the glycome of the female genital tract: cervical-vaginal fluid. PLoS One 11:e0158687. doi:10.1371/journal.pone.0158687 27437931 PMC4954690

[19] Rosen GH, Randis TM, Desai PV, Sapra KJ, Ma B, Gajer P, Humphrys MS, Ravel J, Gelber SE, Ratner AJ. 2017. Group B Streptococcus and the vaginal microbiota. J Infect Dis 216:744–751. doi:10.1093/infdis/jix395 28934437 PMC5853324

[20] Plesniarski A, Siddik AB, Su R-C. 2021. The microbiome as a key regulator of female genital tract barrier function. Front Cell Infect Microbiol 11:790627. doi:10.3389/fcimb.2021.790627 34976864 PMC8719631

[21] Brokaw A, Furuta A, Dacanay M, Rajagopal L, Adams Waldorf KM. 2021. Bacterial and host determinants of group B streptococcal vaginal colonization and ascending infection in pregnancy. Front Cell Infect Microbiol 11:720789. doi:10.3389/fcimb.2021.720789 34540718 PMC8446444

[22] Burcham LR, Burcham ZM, Akbari MS, Metcalf JL, Doran KS. 2022. Interrelated effects of zinc deficiency and the microbiome on group B streptococcal vaginal colonization. mSphere 7:e00264-22. doi:10.1128/msphere.00264-22 35943198 PMC9429885

[23] Derrien M, Vaughan EE, Plugge CM, de Vos WM. 2004. Akkermansia muciniphila gen. nov., sp. nov., a human intestinal mucin-degrading bacterium. Int J Syst Evol Microbiol 54:1469–1476. doi:10.1099/ijs.0.02873-0 15388697

[24] Iwaza R, Wasfy RM, Dubourg G, Raoult D, Lagier J-C. 2022. Akkermansia muciniphila: the state of the art, 18 years after its first discovery. Front Gastroenterol (Lausanne) 1:1024393. doi:10.3389/fgstr.2022.1024393 41822083 PMC12952328

[25] Dao MC, Everard A, Aron-Wisnewsky J, Sokolovska N, Prifti E, Verger EO, Kayser BD, Levenez F, Chilloux J, Hoyles L, MICRO-Obes Consortium, Dumas M-E, Rizkalla SW, Doré J, Cani PD, Clément K. 2016. Akkermansia muciniphila and improved metabolic health during a dietary intervention in obesity: relationship with gut microbiome richness and ecology. Gut 65:426–436. doi:10.1136/gutjnl-2014-308778 26100928

[26] Ottman N, Reunanen J, Meijerink M, Pietilä TE, Kainulainen V, Klievink J, Huuskonen L, Aalvink S, Skurnik M, Boeren S, Satokari R, Mercenier A, Palva A, Smidt H, de Vos WM, Belzer C. 2017. Pili-like proteins of Akkermansia muciniphila modulate host immune responses and gut barrier function. PLoS One 12:e0173004. doi:10.1371/journal.pone.0173004 28249045 PMC5332112

[27] Shen W, Shen M, Zhao X, Zhu H, Yang Y, Lu S, Tan Y, Li G, Li M, Wang J, Hu F, Le S. 2017. Anti-obesity effect of capsaicin in mice fed with high-fat diet is associated with an increase in population of the gut bacterium Akkermansia muciniphila. Front Microbiol 8:272. doi:10.3389/fmicb.2017.00272 28280490 PMC5322252

[28] Zhang T, Li Q, Cheng L, Buch H, Zhang F. 2019*.* Akkermansia muciniphila is a promising probiotic. Microb Biotechnol 12:1109–1125. doi:10.1111/1751-7915.13410 31006995 PMC6801136

[29] Jian H, Liu Y, Wang X, Dong X, Zou X. 2023. Akkermansia muciniphila as a next-generation probiotic in modulating human metabolic homeostasis and disease progression: a role mediated by gut-liver-brain axes? Int J Mol Sci 24:3900. doi:10.3390/ijms24043900 36835309 PMC9959343

[30] Becken B, Davey L, Middleton DR, Mueller KD, Sharma A, Holmes ZC, Dallow E, Remick B, Barton GM, David LA, McCann JR, Armstrong SC, Malkus P, Valdivia RH. 2021. Genotypic and phenotypic diversity among human isolates of Akkermansia muciniphila. mBio 12:e00478-21. doi:10.1128/mBio.00478-21 34006653 PMC8262928

[31] Baud A, Hillion KH, Plainvert C, Tessier V, Tazi A, Mandelbrot L, Poyart C, Kennedy SP. 2023. Microbial diversity in the vaginal microbiota and its link to pregnancy outcomes. Sci Rep 13:9061. doi:10.1038/s41598-023-36126-z 37271782 PMC10239749

[32] Tortelli BA, Lewis AL, Fay JC. 2021. The structure and diversity of strain-level variation in vaginal bacteria. Microb Genom 7:mgen000543. doi:10.1099/mgen.0.000543 33656436 PMC8190618

[33] France MT, Fu L, Rutt L, Yang H, Humphrys MS, Narina S, Gajer PM, Ma B, Forney LJ, Ravel J. 2022. Insight into the ecology of vaginal bacteria through integrative analyses of metagenomic and metatranscriptomic data. Genome Biol 23:66. doi:10.1186/s13059-022-02635-9 35232471 PMC8886902

[34] Jung D-R, Choi Y, Jeong M, Singh V, Jeon SY, Seo I, Park NJ-Y, Lee YH, Park JY, Han HS, Shin J-H, Chong GO. 2025. Metagenomic insight into the vaginal microbiome in women infected with HPV 16 and 18. NPJ Biofilms Microbiomes 11:105. doi:10.1038/s41522-025-00747-1 40506497 PMC12162855

[35] Schlievert PM, Kelly JA. 1984. Clindamycin-induced suppression of toxic-shock syndrome--associated exotoxin production. J Infect Dis 149:471. doi:10.1093/infdis/149.3.471 6715902

[36] Wang N-Y, Patras KA, Seo HS, Cavaco CK, Rösler B, Neely MN, Sullam PM, Doran KS. 2014. Group B streptococcal serine-rich repeat proteins promote interaction with fibrinogen and vaginal colonization. J Infect Dis 210:982–991. doi:10.1093/infdis/jiu151 24620021 PMC4192050

[37] Manzer HS, Nguyen DT, Park JY, Park N, Seo KS, Thornton JA, Nobbs AH, Doran KS. 2022. The group B streptococcal adhesin BspC interacts with host cytokeratin 19 to promote colonization of the female reproductive tract. mBio 13:e01781-22. doi:10.1128/mbio.01781-22 36069447 PMC9600255

[38] Sheen TR, Jimenez A, Wang N-Y, Banerjee A, van Sorge NM, Doran KS. 2011. Serine-rich repeat proteins and pili promote Streptococcus agalactiae colonization of the vaginal tract. J Bacteriol 193:6834–6842. doi:10.1128/JB.00094-11 21984789 PMC3232834

[39] Patras KA, Doran KS. 2016. A murine model of group B Streptococcus vaginal colonization. J Vis Exp:54708. doi:10.3791/54708 27911391 PMC5226234

[40] Li M, Li L, Huang T, Liu Y, Lei A, Ma C, Chen F, Chen M. 2018. Effects of attenuated S. agalactiae strain YM001 on intestinal microbiota of tilapia are recoverable. Front Microbiol 9. doi:10.3389/fmicb.2018.03251 PMC6333689 30687255

[41] Amabebe E, Anumba DOC. 2020. Female gut and genital tract microbiota-induced crosstalk and differential effects of short-chain fatty acids on immune sequelae. Front Immunol 11:2184. doi:10.3389/fimmu.2020.02184 33013918 PMC7511578

[42] El Aila NA, Tency I, Claeys G, Verstraelen H, Saerens B, Santiago GLDS, De Backer E, Cools P, Temmerman M, Verhelst R, Vaneechoutte M. 2009. Identification and genotyping of bacteria from paired vaginal and rectal samples from pregnant women indicates similarity between vaginal and rectal microflora. BMC Infect Dis 9:167. doi:10.1186/1471-2334-9-167 19828036 PMC2770471

[43] Chen Y, Ou Z, Pang M, Tao Z, Zheng X, Huang Z, Wen D, Li Q, Zhou R, Chen P, Situ B, Sheng C, Huang Y, Yue X, Zheng L, Huang L. 2023. Extracellular vesicles derived from Akkermansia muciniphila promote placentation and mitigate preeclampsia in a mouse model. J Extracell Vesicles 12:e12328. doi:10.1002/jev2.12328 37165987 PMC10173384

[44] Liu Y, Qin S, Feng Y, Song Y, Lv N, Liu F, Zhang X, Wang S, Wei Y, Li S, Su S, Zhang W, Xue Y, Hao Y, Zhu B, Ma J, Yang H. 2020. Perturbations of gut microbiota in gestational diabetes mellitus patients induce hyperglycemia in germ-free mice. J Dev Orig Health Dis 11:580–588. doi:10.1017/S2040174420000768 32924908

[45] Yang J, Wang J, Wu W, Su C, Wu Y, Li Q. 2024. Xylooligosaccharides ameliorate insulin resistance by increasing Akkermansia muciniphila and improving intestinal barrier dysfunction in gestational diabetes mellitus mice. Food Funct 15:3122–3129. doi:10.1039/d3fo04681h 38426554

[46] Wang Y, Zhu Y, Cui Y, Fang J, Zhong H, Shi Y, Liu L, Cui X. 2025. Pasteurized Akkermansia muciniphila ameliorates insulin resistance by reducing placental inflammation in GDM mouse model. Reprod Biol 25:101073. doi:10.1016/j.repbio.2025.101073 40912181

[47] Mercado-Evans V, Mejia ME, Zulk JJ, Ottinger S, Hameed ZA, Serchejian C, Marunde MG, Robertson CM, Ballard MB, Ruano SH, Korotkova N, Flores AR, Pennington KA, Patras KA. 2024. Gestational diabetes augments group B Streptococcus infection by disrupting maternal immunity and the vaginal microbiota. Nat Commun 15:1035. doi:10.1038/s41467-024-45336-6 38310089 PMC10838280

[48] Burcham LR, Bath JR, Werlang CA, Lyon LM, Liu N, Evans C, Ribbeck K, Doran KS. 2022. Role of MUC5B during group B streptococcal vaginal colonization. mBio 13:e00039-22. doi:10.1128/mbio.00039-22 35323039 PMC9040740

[49] Song JY, Lim JH, Lim S, Yong Z, Seo HS. 2018. Progress toward a group B streptococcal vaccine. Hum Vaccin Immunother 14:2669–2681. doi:10.1080/21645515.2018.1493326 29995578 PMC6314413

[50] Cieslewicz MJ, Chaffin D, Glusman G, Kasper D, Madan A, Rodrigues S, Fahey J, Wessels MR, Rubens CE. 2005. Structural and genetic diversity of group B streptococcus capsular polysaccharides. Infect Immun 73:3096–3103. doi:10.1128/IAI.73.5.3096-3103.2005 15845517 PMC1087335

[51] Chaffin DO, Beres SB, Yim HH, Rubens CE. 2000. The serotype of type Ia and III group B streptococci is determined by the polymerase gene within the polycistronic capsule operon. J Bacteriol 182:4466–4477. doi:10.1128/JB.182.16.4466-4477.2000 10913080 PMC94618

[52] Shuoker B, Pichler MJ, Jin C, Sakanaka H, Wu H, Gascueña AM, Liu J, Nielsen TS, Holgersson J, Nordberg Karlsson E, Juge N, Meier S, Morth JP, Karlsson NG, Abou Hachem M. 2023. Sialidases and fucosidases of Akkermansia muciniphila are crucial for growth on mucin and nutrient sharing with mucus-associated gut bacteria. Nat Commun 14:1833. doi:10.1038/s41467-023-37533-6 37005422 PMC10067855

[53] Luo Y, Lan C, Li H, Ouyang Q, Kong F, Wu A, Ren Z, Tian G, Cai J, Yu B, He J, Wright A-DG. 2022. Rational consideration of Akkermansia muciniphila targeting intestinal health: advantages and challenges. NPJ Biofilms Microbiomes 8:81. doi:10.1038/s41522-022-00338-4 36253412 PMC9576740

[54] Elzinga J, Narimatsu Y, de Haan N, Clausen H, de Vos WM, Tytgat HLP. 2024. Binding of Akkermansia muciniphila to mucin is O-glycan specific. Nat Commun 15:4582. doi:10.1038/s41467-024-48770-8 38811534 PMC11137150

[55] Fichorova RN, Rheinwald JG, Anderson DJ. 1997. Generation of papillomavirus-immortalized cell lines from normal human ectocervical, endocervical, and vaginal epithelium that maintain expression of tissue-specific differentiation proteins. Biol Reprod 57:847–855. doi:10.1095/biolreprod57.4.847 9314589

[56] Marroquin S, Gimza B, Tomlinson B, Stein M, Frey A, Keogh RA, Zapf R, Todd DA, Cech NB, Carroll RK, Shaw LN. 2019. MroQ is a novel abi-domain protein that influences virulence gene expression in Staphylococcus aureus via modulation of agr activity. Infect Immun 87:e00002-19. doi:10.1128/IAI.00002-19 30833335 PMC6479029

