# Supplementary figures and images for: Akkermansia muciniphila impacts group B Streptococcus vaginal colonization
Source: mBio. 2026 Apr 27;17(6):e02868-25. doi: 10.1128/mbio.02868-25 (PMC13251365; doi:10.1128/mbio.02868-25)

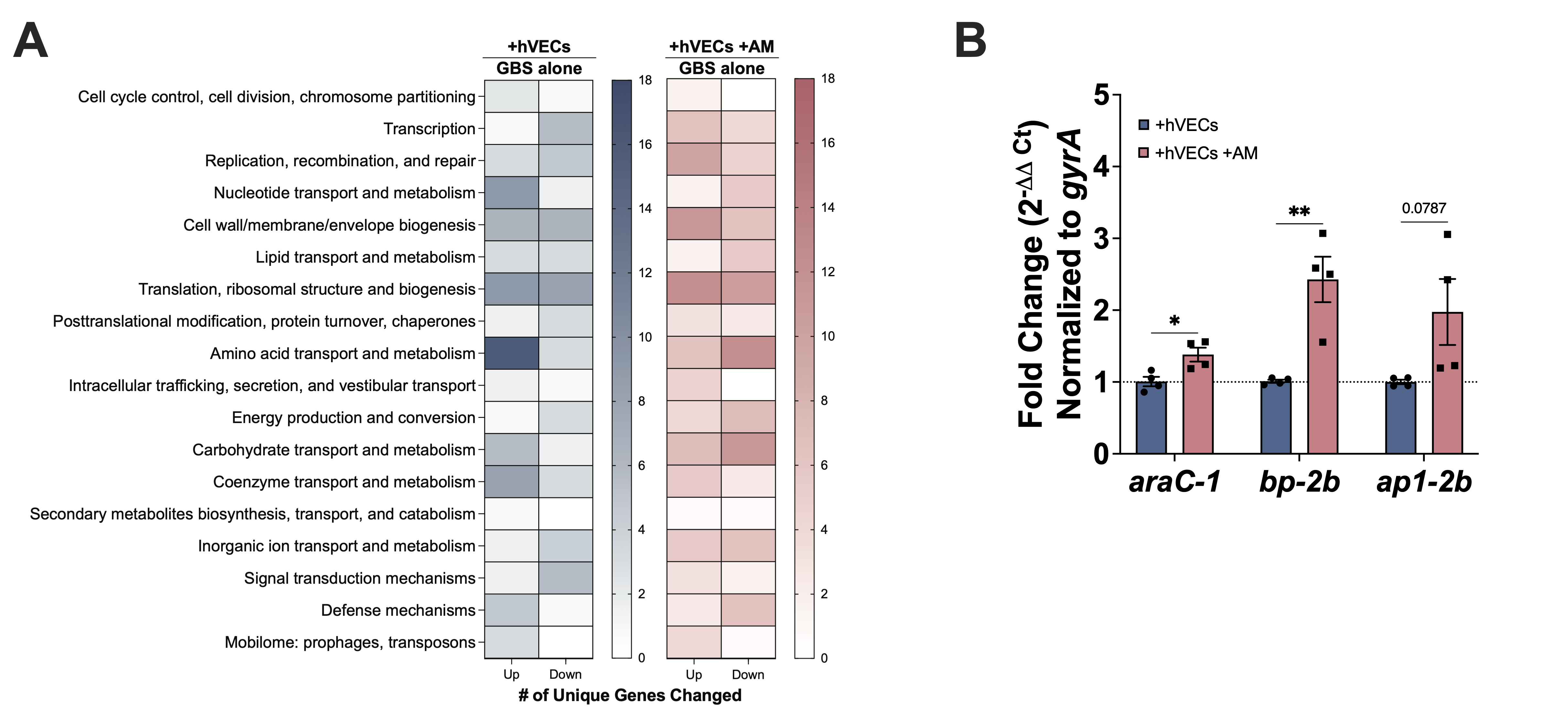

Supplement: Figure S1 — Heat map and RT-qPCR. [file mbio.02868-25-s0001.tiff]
