# Supplementary material for: Akkermansia muciniphila impacts group B Streptococcus vaginal colonization
Source: mBio. 2026 Apr 27;17(6):e02868-25. doi: 10.1128/mbio.02868-25 (PMC13251365; doi:10.1128/mbio.02868-25)
Supplement: Supplemental Material Legends — Figure S1 and Table S1-S3 legends. [file mbio.02868-25-s0002.docx]

**SUPPLEMENTAL TITLES AND LEGENDS**

**Figure S1. Differential expression of GBS biological pathways during mono- and co-infection and validation by RT-qPCR**. **(A)** Differentially expressed genes with an FDR *p*-value ≤ 0.05 and a fold change cut off ≥ |1.5| were sorted based on biological pathways. Data represent three biological replicates. **(B)** RT-qPCR was used to confirm changes observed in PI-1 and PI-2b genes. Statistical analysis was determined using a Student’s T-test. Data represent technical duplicates from two independent experiments. Error bars represent +/- SEM. *, *p* ≤ .05; **, *p* ≤ .01.

**Table S1. All differentially expressed GBS genes with a ≥ |1.5|-fold change and an FDR *p*-value ≤ 0.05 in mono-infection (GBS+hVECs) and co-infection (GBS+AM+hVECs) using GBS alone (grown in KSFM) as a control.** Excel table.

**Table S2. All differentially expressed GBS genes with a ≥ |1.5|-fold change and an FDR *p*-value ≤ 0.05 in co-infection (GBS+AM+hVECs) using mono-infection (GBS+hVECs) as a control.** Excel table.

**Table S3. Sequencing and mapping statistics for RNAseq analysis.** Excel table.
